# p53 induces senescence through Lamin A/C stabilization-mediated nuclear deformation

**DOI:** 10.1038/s41419-019-1378-7

**Published:** 2019-02-06

**Authors:** Min-Ho Yoon, So-mi Kang, Su-Jin Lee, Tae-Gyun Woo, Ah-Young Oh, Soyoung Park, Nam-Chul Ha, Bum-Joon Park

**Affiliations:** 1 Department of Molecular Biology, College of Natural Science, Pusan National University, Busan, 46241 Republic of Korea; 2 Department of Food Science, College of Agricultural Science, Seoul National University, Seoul, 08826 Republic of Korea

## Abstract

p53-mediated cellular senescence has been intensively investigated, because it is important for tumor suppressive function. In addition, p16/INK4A is well known to be critical for cellular senescence. However, detailed molecular mechanism or relevance between p53 and p16-mediated senescence has not been demonstrated yet. Here we show that p53 induces p16 through Lamin A/C stabilization via direct interaction. Stabilized Lamin A/C promotes degradation of BMI-1 and MEL-18 (Polycomb repressor complex 1, PRC1), which sequesters p16 promotor. Increased p53 can reduce BMI-1/MEL-18 and induce p16 expression via Lamin A/C. Elimination of Lamin A/C can abolish p53-induced p16 expression and BMI-1/MEL-18 reduction. As Lamin A/C expression is increased during cell differentiation, this mechanism seems to be very useful for selective induction of senescence in non-stem cells. Our results suggest that Lamin A/C-p53 network is important for p16/INK4A-mediated cellular senescence.

## Introduction

Lamin A/C is an intermediated filament protein that forms the inner nuclear membrane architecture. Its expression is detected when cells are differentiated^1^. Aberrant splicing product of Lamin A termed progerin (PRG) is the causal protein of premature senescence in Hutchinson–Gilford Progeria syndrome (HGPS)^2,3^. The characteristic feature of HGPS cells is nuclear deformation, suggesting that deregulation of nuclear architecture or integrity might be an important cause of cellular senescence^4,5^. Considering that Lamin A/C expression is coupled with cell differentiation while stem cells do not express Lamin A/C, increase in Lamin A/C expression might be related to the initiation of cellular aging^6,7^.

p53 has also been suggested as an important cellular senescence inducer. p53-induced cellular senescence is known to be an important and primary tumor suppressive barrier^8–11^. Concerning the relevance between p53 and senescence, there are many conflicting results. Some p53 transgenic mouse models such as N-terminal mutant mouse^12^ show obviously premature aging phenotype^13–15^. In contrast, “super-p53” or hypomorphic MDM2 mice do not display aging-related phenotypes despite elevated p53 expression^16,17^. Recently, it has been reported that mutation of MDM2, which does not suppress p53 expression, is a casual defect in Werner-like segmental progeriod syndrome^18^. This result strongly suggests that deregulation of p53 can induce aging-related features.

Another well-confirmed aging-related protein is p16/INK4A. It is induced in aged cells^19–21^. Overexpression of p16/INK4A can promote cellular senescence^22,23^. Recent studies have reported that elimination of p16/INK4A-expressed cells via cell-suicide system can extend the life span of mice^24–26^. It has been well demonstrated that p53-induced senescence is coupled with p16/INK4A induction^22,27^. However, detailed molecular mechanism regarding p16 induction under p53-induced senescent condition is not well understood yet. In this study, we found that transcriptional activity of p53 was not essential for senescence. Instead, stabilization of p53 itself is required for Lamin A/C induction at posttranslational level. Elevated Lamin A/C induced nuclear deformation and reduction of BMI-1/MEL-18 (components of the Polycomb repressor complex 1, PRC1). As a result of destabilization of PRC1, p16 expression was increased and cellular senescence was accomplished. In fact, elimination of Lamin A/C blocked p53-induced senescence and p16 expression. Our results indicate that stabilization of p53 without transcriptional activation is sufficient for p16-mediated cellular senescence via Lamin A stabilization.

## Results

### p53 induces HGPS-like nuclear deformation

**Fig. 1:**
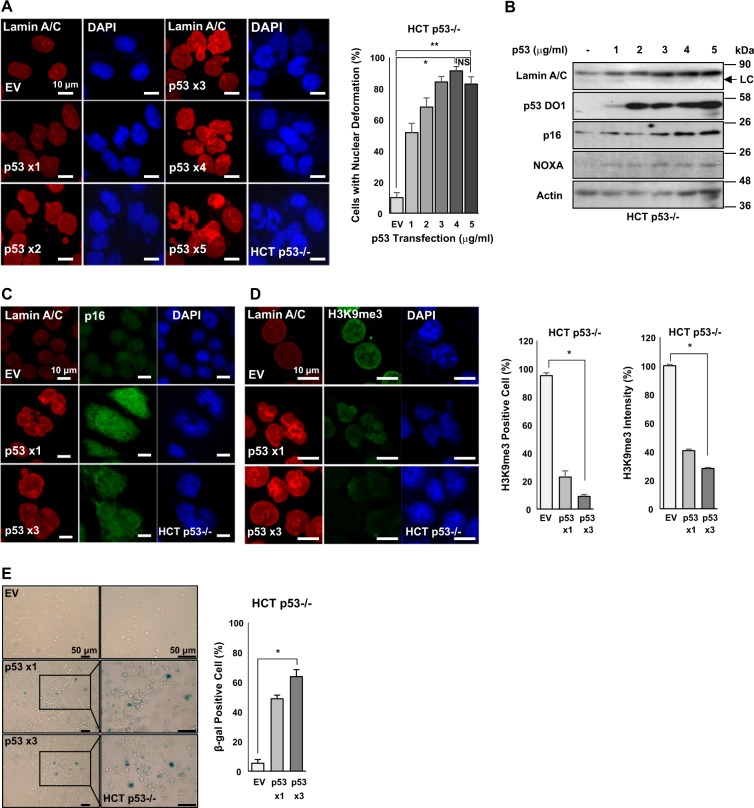
p53 overexpression induces nuclear deformation, Lamin A/C expression, and p16 expression. **a** p53 overexpression induces nuclear deformation. Immunofluorescence (IF) images showing nuclear deformation through dose-dependent p53 transfection (1–5 μg/ml, 48 h). p53-negative HCT116 (HCT p53−/−) cells were transfected with different doses of p53 followed by IF staining (left). Nuclear deformation rate was calculated based on IF images (right). *P < 0.05, **P < 0.001, NS, not significant. **b** Elevated endogenous Lamin A/C expression is dependent on different doses of p53 transfection (1–5 μg/ml, 48 h) in HCT p53−/− cells. Senescence marker p16 was increased by p53 transfection. p53 target gene *NOXA* was also induced by p53 transfection. Actin was used as loading control. Western blotting data of three independent experiments are shown. Lower and weak bands in Lamin A/C blot are Lamin C (LC). **c** p53 overexpression increases p16 expression. Immunofluorescence images of nuclear deformation and p16 expression in HCT p53−/− cells are shown. Cells were transfected with different doses of p53 (1–3 μg/ml, 48 h). IF staining was then performed using Lamin A/C (Red), p16 (Green), and counterstaining using DAPI (Blue). **d** p53 overexpression decreases H3K9me3 expression. IF images of nuclear deformation and histone H3K9me3 expression in HCT p53−/− cells (left) are shown. Counting of histone H3K9me3-positive cell (middle) and signal intensities (right) based on IF staining. Cells were transfected different doses of p53 (1–3 μg/ml, 48 h). IF staining was then performed using Lamin A/C (Red), H3K9me3 (Green), and counterstaining using DAPI (Blue). *P < 0.05. **e** p53 overexpression increases cellular senescence. SA-β-gal staining showed that dose-dependent p53 transfection (1, 3 μg/ml, 48 h) increased the senescence in p53-null cells (left). Counting of β-gal-positive cells (right) is shown. Boxes indicate magnified regions displayed in the right panel. *P < 0.05

HGPS-like nuclear deformation in normal aging process has been reported^2,28^. Therefore, nuclear deformation might be a general feature of cellular aging, particularly p53-induced cellular senescence. To address this possibility, we transfected wild-type p53 into p53-deficient HCT116 (HCT p53−/−) cells. Our results showed that the number of abnormal nuclear cells was increased by p53 transfection (Fig. 1a, b and Supplementary Fig. 1). In addition, inner nuclear membrane proteins Lamin A/C and p16/INK4A, an important senescence marker^21,23^, were induced (Fig. 1b). The induction of p16/INK4A was also confirmed by immunofluorescence (IF) staining (Fig. 1c). In addition, H3K9me3, another senescence marker^2,5^, was clearly reduced in p53-transfected cells (Fig. 1d). In fact, the number of H3K9me3-expressed cells and the intensity of H3K9me3 expression were decreased by p53 transfection (Fig. 1d). Expression of senescence-associated β-galactosidase (SA-β-gal), a more common senescence marker, was also induced by p53 overexpression (Fig. 1e). These results indicate that p53-induced senescence is associated with nuclear deformation and p16 induction.

### Increase of endogenous p53 induces cellular senescence

**Fig. 2:**
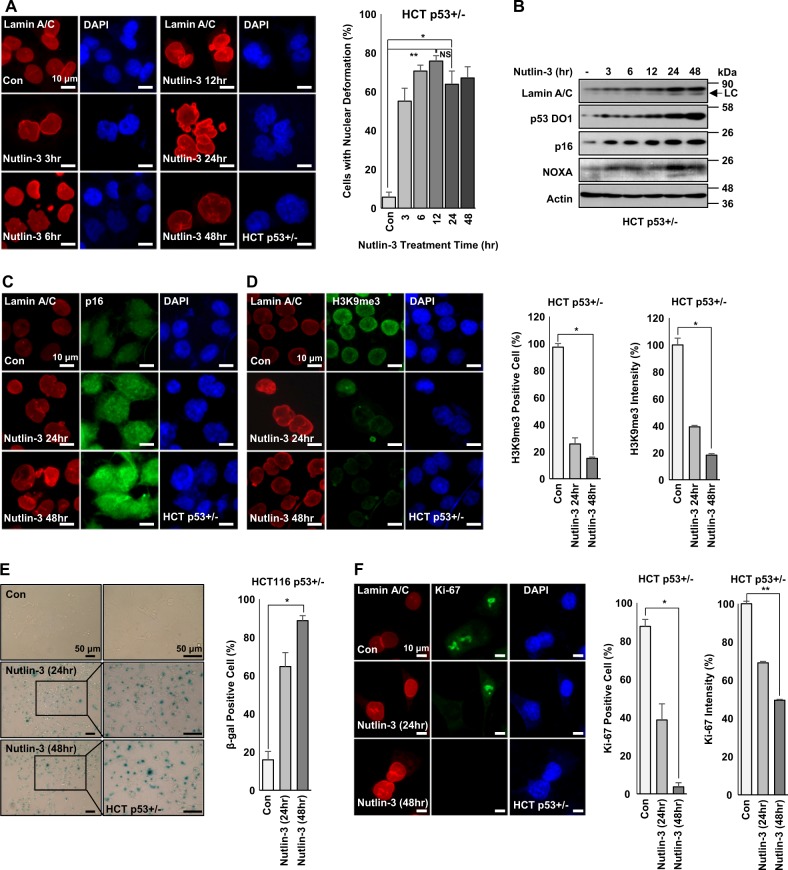
p53 stabilization induces nuclear deformation, Lamin A/C expression, and p16 expression. **a** Nutlin-3 induces nuclear deformation in parental HCT116 (HCT p53+/−) cells in a time-dependent manner (left). Nuclear deformation rate by Nutlin-3 was counted (left). HCT p53+/− cells were treated with Nutlin-3 (1 μM) for different time (24, 48 h). After treatment, cells were IF stained with Lamin A/C (Red) and counterstained with DAPI (Blue). *P < 0.05, **P < 0.03, NS, not significant. **b** Western blotting image shows that stabilized p53 by Nutlin-3 treatment increases expression of Lamin A/C, p16, and NOXA. HCT p53+/− cells were treated with Nutlin-3 (1 μM) for different time period (3–48 h). After treatment, cell extracts were analyzed by western blotting. Actin was used as loading control. Western blotting data of three independent experiments are shown. Lower and weak bands in Lamin A/C blot are Lamin C (LC). **c** Immunofluorescence images of nuclear deformation and p16 expression after Nutlin-3 treatment for different time period. HCT p53+/− cells were treated with Nutlin-3 (1 μM) for different time (24, 48 h). After treatment, cells were subjected to immunofluorescence staining for Lamin A/C (Red), p16 (Green), and counterstained with DAPI (Blue). **d** Immunofluorescence images of nuclear deformation and decreased expression of H3K9me3 in HCT p53+/− cells (left). Counting of histone H3K9me3-positive cells (middle) and signal intensities (right) on the basis of IF staining. HCT p53+/− cells were treated with Nutlin-3 (1 μM) for different time (24, 48 h). After treatment, cells were IF stained with Lamin A/C (Red), H3K9me3 (Green), and counterstained with DAPI (Blue). *P < 0.05. **e** p53 stabilization increases cellular senescence. SA-β-gal staining shows Nutlin-3 treatment increased senescence in p53-positive cell (left) in a time-dependent manner. Counting of β-gal-positive cells (right). HCT p53+/− cells were treated with Nutlin-3(1 μM) for different time (24, 48 h). After treatment, cells were stained with SA-β-gal. Boxes indicate magnified regions displayed in the right panel. *P < 0.03. **f** p53 stabilization decreases cell proliferation. Ki-67 staining is reduced after p53 stabilization for accompanying cells with nuclear deformation (left). Counting of Ki-67-positive cell (middle) and signal intensity (right) on the basis of IF staining. HCT p53+/− cells were treated with Nutlin-3 (1 μM) for different time (24, 48 h) followed by IF staining using Lamin A/C (Red), Ki-67 (Green), and counterstaining using DAPI (Blue). *P < 0.05, **P < 0.03

To address the effect of endogenous p53 stabilization, we treated p53-positive HCT116 cells^29^ with Nutlin-3 (Nut), an inhibitor of p53-MDM2 binding, and monitored nuclear deformation and senescence. Treatment with Nut induced nuclear deformation in a time-dependent manner (Fig. 2a). In Nut-treated HCT116 cells, p16/INK4A and Lamin A/C were also induced (Fig. 2b). The induction of p16/INK4A and the reduction of H3M9me3 were confirmed by IF staining (Fig. 2c, d). However, Nut treatment did not lead to nuclear deformation or induction of p16/INK4A or Lamin A/C in HCT116 p53−/− cells (Supplementary Figs. 2A-2C). After Nut treatment, SA-β-gal was induced in p53-positive HCT116, but not in p53−/− HCT116 cells (Fig. 2e and Supplementary Fig. 2D). In fact, Nut induced growth arrest in p53-positive cell lines (Supplementary Fig. 3A). Level of proliferation marker Ki-67 was also decreased in p53-positive HCT116 cells (Fig. 2f and Supplementary Fig. 3B), supporting that p53 stabilization could induce cellular senescence. To determine whether Nut could only induce senescence in p53-positive cells, changes in cellular morphology were observed. Spreading out and increased cytoplasmic area are well-known features of senescent cells^30^. To measure cytoplasm area and spreading out, cells were stained with Phalloidin (cytoskeleton) and Paxillin (focal adhesion). Nut induced cytoplasmic spreading (Supplementary Fig. 4A) and p16 expression in p53-positive HCT116 cells (Supplementary Fig. 4B). Indeed, most cells showed spread morphology. The occupied area was increased up to 80% (Supplementary Fig. 4C). In contrast, p53-deficient cells did not show similar morphological changes after Nut treatment (Supplementary Fig. 4D-F). These results strongly suggest that p53 stabilization can induce typical cellular senescence.

Although p53 induction evokes cellular senescence, p53 is also involved in apoptosis. During cell death, nuclear morphological changes are known to occur. Hence, we analyzed cell death under our experimental conditions. Our results showed that treatment with Nut did not evoke any viability change in HCT116 cell lines regardless of p53 status (Supplementary Fig. 5A). Moreover, fluorescence-activated cell sorting analysis revealed that Nut treatment did not lead to cell death in p53 wild-type (HCT p53+/−) or -deficient (HCT p53−/−) cells (Supplementary Fig. 5B, 5D). The irrelevance of apoptosis in our experimental conditions was confirmed through Annexin V staining (Supplementary Fig. 5C).

### DNA damage induces nuclear deformation and p16 expression in a p53-dependent manner

Next, we checked the effect of other p53 inducers on nuclear deformation. First, we blocked p53 degradation using MG132, a general proteasome inhibitor. Treatment with MG132 did not induce nuclear deformation (Supplementary Fig. 6A) or expression of Lamin A/C or p16/INK4 (Supplementary Fig. 6B), despite an obvious increase of p53 (Supplementary Fig. 6B). This implies that ubiquitin-conjugated p53 does not induce senescence. However, as a physiological aging stimulus, p53 activated by UV-irradiation induced nuclear deformation (Supplementary Fig. 6C and 6D) and expression of p16/INK4A and Lamin A/C (Supplementary Fig. 6E). We did not observe nuclear deformation in p53-deficient HCT116 cells (Supplementary Fig. 6F and 6G) under the same UV-irradiation condition. These results suggest that DNA damage-induced cellular senescence is also mediated by p53.

### Transcription activity of p53 is dispensable for nuclear deformation

**Fig. 3:**
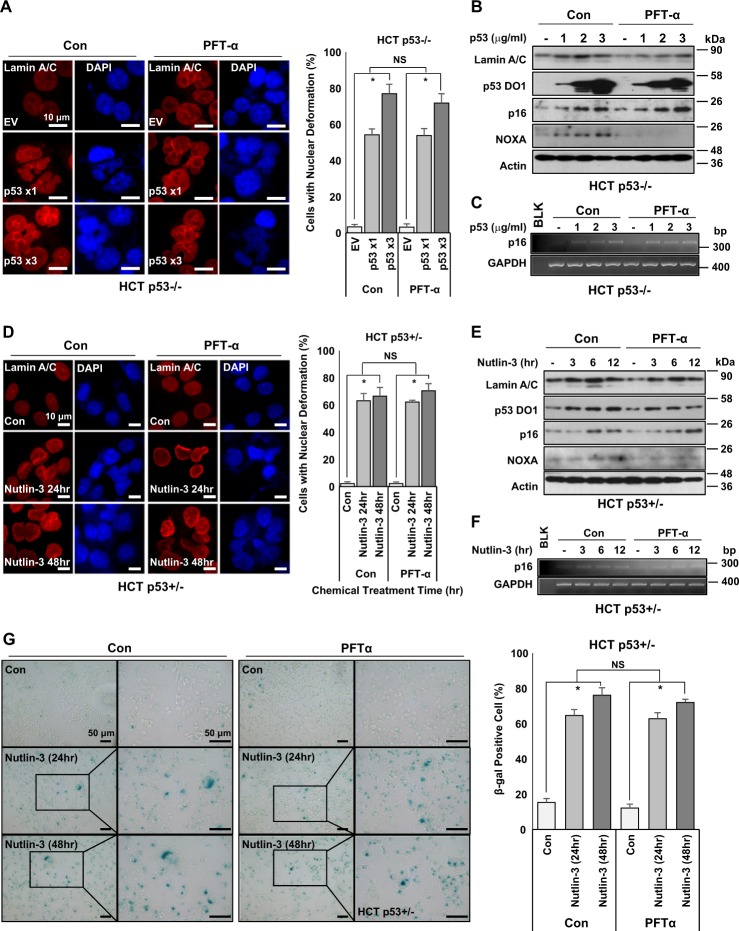
Transcription activity of p53 is dispensable for nuclear deformation. **a** Immunofluorescence images showing p53 causes nuclear deformation with or without PFT-α treatment. PFT-α, a p53 transcription inhibitor, does not affect p53-dependent nuclear deformation (left). Cells with nuclear deformation were counted (right). HCT p53−/− cells were transfected with different doses (1, 3 μg/ml, 48 h) of p53. After transfection, cells were treated with PFT-α (20 μM) for 24 h. After that, cells were stained with Lamin A/C (Red) and counterstained with DAPI (Blue). *P < 0.05, NS, not significant. **b** Western blotting images depicting that PFT-α does not affect p53-dependent Lamin A/C or p16 expression. However, it did affect p53 transcriptional target gene *NOXA*. HCT p53−/− cells were transfected with several doses (1–3 μg/ml) of p53 for 48 h and then treated with PFT-α (20 μM) for 24 h. Cell extracts were used for western blotting using specific antibodies. **c** RT-PCR images showing that p16 upregulation is independent of p53 transcriptional activity. p16 expression at transcriptional level, but not its transcriptional activity, was associated with p53 quantity. HCT p53−/− cells were transfected several doses (1–3 μg/ml) of p53 for 48 h and treated with PFT-α (20 μM) for 24 h. RT-PCR was then performed. **d** Immunofluorescence images showing that stabilized p53 causes nuclear deformation with or without PFT-α treatment in HCT p53+/− cells. Transcriptional inhibition of p53 does not affect nuclear deformation caused by p53 stabilization (left). Nuclear deformation rates were counted (right). HCT p53+/− cells were treated with Nutlin-3 (1 μM) for different time periods (24, 48 h) with or without PFT-α (20 μM). After chemical treatment, cells were stained with Lamin A/C (Red) and counterstained with DAPI (Blue). *P < 0.05, NS, not significant. **e** Western blotting images showing p53 transcriptional activity is independent of p16 induction. Stabilized p53 elevated Lamin A/C and p16 regardless of PFT-α treatment. Inhibition of p53 transcriptional activity only affected NOXA expression. HCT p53+/− cells were treated with Nutlin-3 (1 μM) for different time periods (24, 48 h) with or without PFT-α (20 μM). After chemical treatment, cell extracts were subjected to western blotting using specific antibodies. **f** RT-PCR images depicting that p16 expression caused by p53 stabilization is independent of PFT-α. HCT p53+/− cells were treated with Nutlin-3 (1 μM) for different time periods (24, 48 h) with or without treatment with PFT-α (20 μM). RT-PCR was then performed. GAPDH is a loading control. **g** p53 stabilization induces cellular senescence independent of PFT-α. SA**-**β-gal staining showed that Nutlin-3 (1 μM, 24, 48 h) induced senescence regardless of treatment with PFT-α (20 μM, 24 h) (left). Results of counting of β-gal-positive cells (right) are shown. Boxes indicate magnified regions displayed in the right panel. *P < 0.05, NS, not significant

As the main function of p53 is transcription factor, the requirement of its transcriptional activity on nuclear deformation is tested next. Pifithrin-α (PFT-α)^31^, p53 transcription inhibitor, did not block p53-induced nuclear deformation (Fig. 3a) or p16/INK4A expression (Fig. 3b), although it completely inhibited the expression of *NOXA*, a direct transcriptional target gene^32^. Increase of p16/INK4 mRNA was detected in PFT-α-treated cells, although p53 target genes such as *p21*, *Bax*, *Puma*, and *Noxa* were completely inhibited (Fig. 3c and Supplementary Fig. 7A). Similar results were also obtained after Nut treatment. Regardless of treatment with PFT-α, Nut induced nuclear deformation (Fig. 3d) and p16/INK4A expression (Fig. 3e). Induction of p16/INK4A at transcription level by Nut regardless of PFT-α treatment was also confirmed by reverse transcription PCR (RT-PCR) (Fig. 3f and Supplementary Fig. 7B-D). SA-β-gal staining showed that PFT-α had no effect on cell senescence (Fig. 3g). We also observed the dispensability of p53 transcriptional activity for senescence in other p53 wild-type cell lines. Indeed, we could induce nuclear deformation by treatment with Nut in A549 (a human lung cancer cell line) and MCF-7 (a human breast cancer cell line) regardless of PFT-α treatment (Supplementary Figs. 8A-8D). Therefore, we checked nuclear morphology under p53-activated conditions. Treatment with Etoposide (Etop), although it can induce apoptosis, also induced nuclear deformation in HCT116 cells (Supplementary Figs. 8E and 8F). In addition, treatment with Adriamycin (Adr) induced p16/INK4A expression despite PFT-α treatment (Supplementary Fig. 8G). However, Nut treatment did not induce nuclear deformation in WI-26 (SV40 Large T antigen-expressed lung fibroblasts; Supplementary Fig. 9A and 9B) or Capan-1 (p53-mutated pancreatic cancer cell line; Supplementary Fig. 9C and 9D). These results indicate that wild-type p53 is required for nuclear deformation.

### Major p53 target genes are not involved in senescent phenotype

PTF-α is widely used as a p53 transcription inhibitor^33^. Our results did not completely exclude the engagement of p53 target genes in senescence. Thus, we checked the effect of Nut on three kinds of major p53 target gene-deleted HCT116 isogenic cell lines: HCT p21−/−, HCT Bax−/−, and HCT Puma−/−. In these cell lines, Nut induced nuclear deformation and Lamin A/C expression (Supplementary Figs. 10A, 10C, and 10E). p16/INK4A was also significantly increased by Nut treatment (Supplementary Figs. 4B, 4D, and 4F). Our results suggested that major p53 target genes involved in cell cycle arrest (p21) and apoptosis (Bax and PUMA) are not critical for p53-induced senescence.

### p16/INK4A is essential for p53-induced senescence

**Fig. 4:**
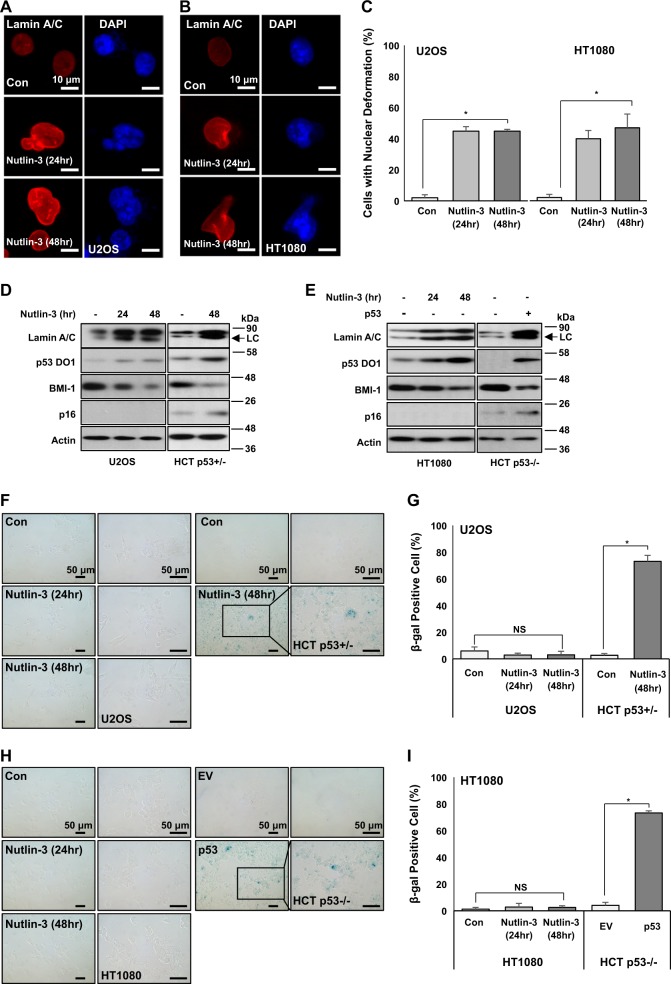
p53-induced senescence via Lamin A relies on p16 expression. **a** Nuclear deformation by p53 stabilization in p16 transcriptionally repressed U2OS cells. Although p16 expression was repressed, p53 stabilization by Nutlin-3 induced nuclear deformation. U2OS cells were treated with Nutlin-3 (1 μM) for different time periods (24 and 48 h). After treatment, cells were IF stained with Lamin A/C (Red) and counterstained with DAPI (Blue). **b** Nuclear deformation by p53 stabilization in p16-null HT1080 cells. p53 stabilization by Nutlin-3 induced nuclear deformation in p16-deficient cells. HT1080 cells were treated with Nutlin-3 (1 μM) for different time periods (24, 48 h). After treatment, cells were IF stained with Lamin A/C (Red) and counterstained with DAPI (Blue). **c** Cells with nuclear deformation were counted in **a** and **b**. *P < 0.01. **d** Stabilized p53 increases Lamin A/C expression and inhibits BMI-1 expression. p53 stabilization by Nutlin-3 increased Lamin A/C expression but decreased BMI-1 expression in p16-repressed U2OS cells. Cells were treated with Nutlin-3 (1 μM) for different time periods (24 and 48 h). After treatment, cell extracts were analyzed by western blotting using specific antibodies. HCT p53+/− was a positive control for p16 expression. Lower and weak bands in Lamin A/C blot are Lamin C (LC). **e** p53 stabilization elevates Lamin A/C expression and inhibits BMI-1 expression in p16-deficient cells. p53 stabilization by Nutlin-3 elevated Lamin A/C expression but decreased BMI-1 expression in p16-null HT1080 cells. As a positive control, only p53-transfected HCT p53−/− cells showed p16 induction. HT1080 cells were treated with Nutlin-3 (1 μM) for different time periods (24 and 48 h). HCT p53−/− cells were transfected with p53 (1.5 μg/ml) for 48 h. After treatment and transfection, cell extracts were analyzed by western blotting using specific antibodies. Lower and weak bands in Lamin A/C blot are Lamin C (LC). **f** Lamin A/C expression increased by p53 stabilization does not induce cellular senescence in p16 expression-repressed U2OS cells. After treatment with Nutlin-3 for different time periods, U2OS cells did not show cellular senescence phenotype. As positive control, only Nutlin-3-treated HCT p53+/− cells showed β-gal-positive staining. U2OS and HCT p53+/− cells were treated with Nutlin-3 (1 μM and 24, 48 h). After treatment, cells were subjected to SA-β-gal staining. Boxes indicate magnified regions displayed in the right panel. **g** Counting of β-gal-positive cells in **f**. *P < 0.01, NS, not significant. **h** Lamin A/C expression increased by p53 stabilization does not induce cellular senescence in p16-null HT1080 cells. After treatment with Nutlin-3 for different time periods, HT1080 cells did not show cellular senescence phenotype. As positive control, only p53-transfected HCT p53−/− cells showed β-gal-positive staining. HT1080 cells were treated with Nutlin-3 (1 μM) for different time periods (24 and 48 h). HCT p53−/− cells were transfected with p53 (1.5 μg/ml, 48 h). After treatment and transfection, cells were subjected to SA-β-gal staining. Boxes indicate magnified regions displayed in the right panel. **i** Counting of β-gal-positive cells in **h**. *P < 0.01, NS, not significant

We next investigated the role of p16/INK4A in p53-induced senescence using p16/INK4A-deficient cell lines (U2OS and HT1080)^34,35^. Stabilization of p53 by Nut induced Lamin A/C expression (Fig. 4a, b) and nuclear deformation (Fig. 4c) in both cell lines. Western blotting analysis also showed the induction of Lamin A/C and p53 expression in both cell lines (Fig. 4d, e). However, expression of SA-β-gal, a general senescence marker, was not increased in either cell line (Fig. 4f–h), despite its obvious increase in Nut-treated HCT116 p53+/− and p53-transfected HCT116 p53−/− cells (Fig. 4g–i). These results imply that nuclear deformation can be achieved by p53-Lamin A network and p16/INK4 is a final effector of senescence.

**Fig. 5:**
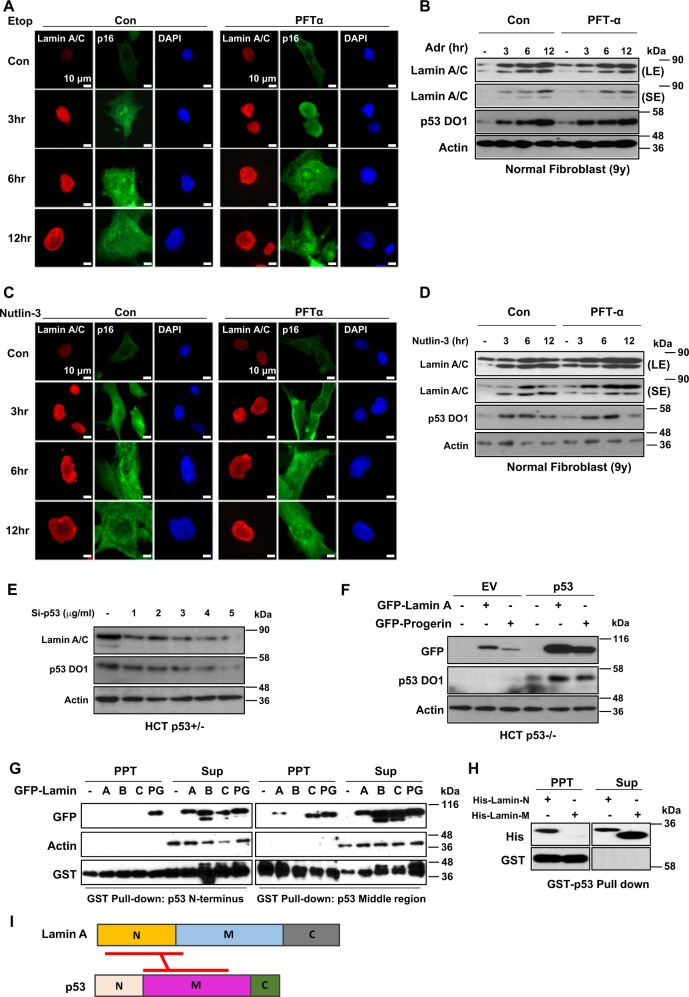
Wild-type p53 stabilizes Lamin A/C via direct interaction. **a** DNA damage increases nuclear deformation and expression of p53 and Lamin A/C with or without PFT-α treatment. Etoposide (Etop)-induced nuclear deformation is not affected by PFT-α. Normal fibroblast cells (N9) were treated with Etop (10 μM) for different time periods (3, 6, and 12 h) with or without treatment with PFT-α (20 μM). Cells were stained with Lamin A/C (Red), p16 (Green), and counterstained with DAPI (Blue). **b** Adriamycin (Adr) induces p53 expression that in turn upregulates Lamin A/C and p16 regardless of PFT-α treatment. N9 cells were treated with Adr (2 μg/ml) for different time periods (3, 6, and 12 h) with or without treatment with PFT-α (20 μM). Cell extracts were analyzed by western blotting with specific antibodies. LE and SE indicated long exposure and short exposure, respectively. **c** Nutlin-3 induces nuclear deformation regardless of PFT-α treatment. N9 cells were treated with Nutlin-3 (1 μM) for different time periods (3, 6, and 12 h) with or without treatment with PFT-α (20 μM). Cells were stained with Lamin A/C (Red), p16 (Green), and counterstained with DAPI (Blue). **d** Western blotting analyses revealed increase of Lamin A/C expression by stabilized p53 with or without treatment with PFT-α. Nutlin-3 stabilizes p53, which upregulates Lamin A/C and p16. PFT-α did not affect the increase of p16 expression. N9 cells were treated with Nutlin-3 (1 μM) for different time periods (3, 6, and 12 h) with or without treatment with PFT-α (20 μM). Cell extracts were analyzed by western blotting with specific antibodies. LE and SE indicated long exposure and short exposure, respectively. **e** Western blotting depicts that Lamin A/C expression is dependent on p53 expression. Small interference RNA (Si-RNA) of p53 knockdowns endogenous p53 expression in HCT p53+/− cells in a dose-dependent manner. Decreased p53 expression affects Lamin A/C downregulation. Cells were transfected with several doses (1–5 μg/ml) of Si-p53 for 48 h. After transfection, cell extracts were analyzed with western blotting with specific antibodies. **f** Western blotting analyses of elevated exogenous Lamin A and progerin expression by p53. Co-transfected p53 upregulates GFP-tagged Lamin A and progerin (GFP-Lamin A, GFP-Progerin). Vectors (1.5 μg/ml) were transfected in HCT p53−/− cells (24 h) and cell extracts were analyzed by western blotting. **g** Pulldown assay for p53 and Lamin isotypes. p53 middle region binds to Lamin A, while progerin binds to both p53 N-terminal and p53 middle regions. The letters (**a**, **b**, **c**, and PG) in GFP-Lamin indicated Lamin A, Lamin B, Lamin C, and progerin, respectively. PPT and Sup indicated precipitated materials and Supernatant of GST-pulldown, respectively. **h** Binding of p53 and Lamin A. GST-pulldown assay shows that p53 binds with N-terminal region of Lamin A more than middle region. Recombinant Lamin A proteins (His-Lamin-N; 1–300 AA, His-Lamin-M; 240–400 AA) were incubated with bead-conjugated p53 core protein (1 h). After incubation, samples were separated and analyzed by western blotting. *PPT* Pellet, *Sup* Supernatant. **i** Scheme for binding region of Lamin A and p53. Lamin A N-terminal binds with middle region of p53

As we used human cancer cell lines, we wondered whether p53-mediated Lamin A/C induction was a physiological pathway. To address this, we treated human normal fibroblasts (obtained from a 9-year-old child) with p53 activators such as Etop and Nut. Nuclear deformation and Lamin A/C expression were then monitored. In normal fibroblasts, we could observe the induction of Lamin A/C and p16/INK4A in response to treatment with Etop (Fig. 5a) and Adr (Fig. 5b). Treatment with Nut also induced Lamin A/C and p16/INK4A expression (Fig. 5c, d) following p53 stabilization. However, PTF-α did not block senescence or reduction of H3K9me3 (Supplementary Figs. 11A and 11B). Our results showed that p53-mediated Lamin A/C induction would be one of general senescence pathways.

### Wild-type p53 stabilizes Lamin A/C via direct interaction

Next, we investigated how p53 induced Lamin A/C expression. Transfection of p53 into PC3 (p53-null prostate cancer cell line) induced Lamin A/C expression (Supplementary Fig. 11C) without transcriptional induction (Supplementary Fig. 11D), indicating that Lamin A/C induction could be achieved at posttranscriptional level. In contrast, elimination of p53 reduced Lamin A/C expression (Fig. 5e and Supplementary Figs. 11E and 11F). In fact, transfection of p53 induced ectopic expression of Lamin A/C and PRG (Fig. 5f). To explore the molecular mechanism involved in p53-mediated Lamin A/C induction, we first tested direct interaction between them. Through glutathione *S*-transferase (GST)-pulldown assay, we found that p53 core domain (p53 Middle region) was responsible for binding to Lamin A/C and PRG, but not Lamin B (Fig. 5g and Supplementary Fig. 11G). This result showed that the binding between p53 and Lamin A/C was specific event and mediated by common region of Lamin A/C and PRG. Additional binding between PRG and p53 N-terminal region was also found (Fig. 5g). This could be related to more obvious nuclear deformation pattern in HGPS cells. We also observed direct interaction of N-terminal Lamin A (1–300 AA) and p53 through in vitro binding assay (Fig. 5h). Overexpression of SV40 Large T antigen known to bind to p53 core domain^36,37^ interrupted the interaction between p53 and Lamin A (Supplementary Figs. 11H and 11I). This supports our hypothesis that N-terminal domain of Lamin A can directly interact with p53 core domain (Fig. 5i). Indeed, SV40 Large T antigen suppressed the expression of Lamin A and PRG (Supplementary Fig. 11J).

### Lamin A/C induces p16/INK4A and senescence

**Fig. 6:**
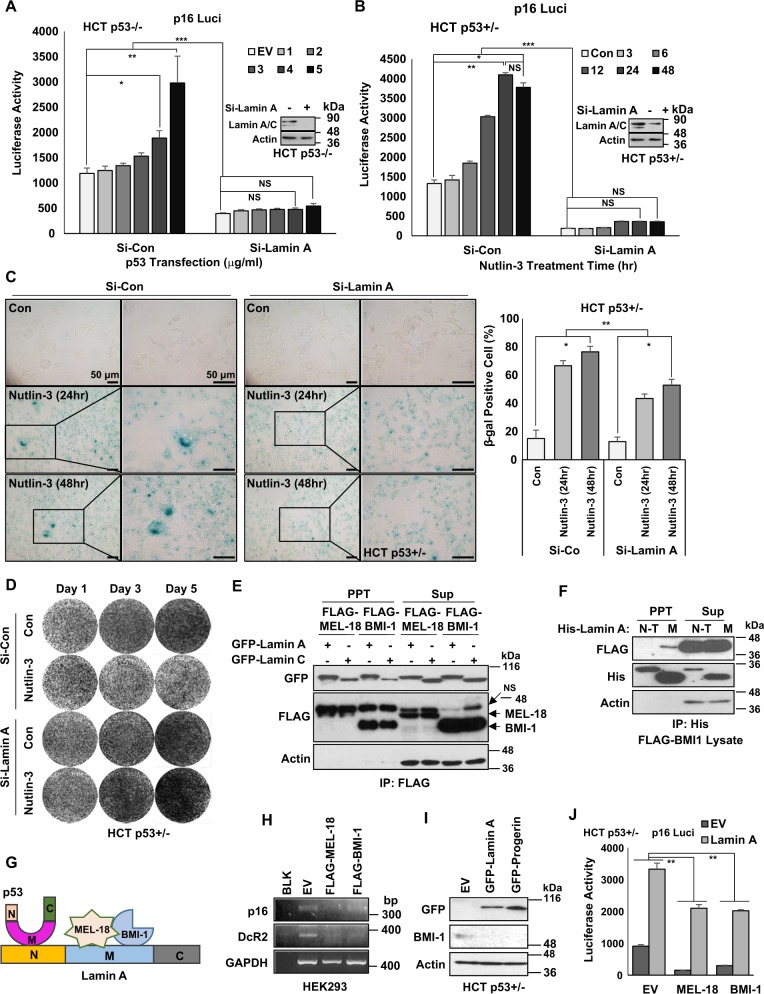
Lamin A/p53 induce p16 via BMI-1/MEL-18. **a** Knockdown of Lamin A blocked p53-mediated p16/INK4A promotor activation. Elimination of Lamin A via Si-Lamin A (inset) abolished the induction of p16/INK4 promotor activity in response to p53 transfection (indicated concentration; μg/ml) for 24 h into HCT p53−/− cells. **b** Si-Lamin A (inset) abrogates Nutlin-3-induced p16/INK4A promotor activity. HCT116 cells were incubated with 1 μM of Nutlin-3 for indicated time (h). *P < 0.05, **P < 0.03, ***P = 0.001, NS, not significant. **c** p53-induced cellular senescence is suppressed by Lamin A knockdown. SA-β-gal staining shows that Lamin A knockdown decreases senescence (left). Counting of β-gal-positive cells (right). p53-positive HCT p53+/− cells were transfected with Si-Lamin A (2.5 μg/ml, 48 h) and treated with Nutlin-3 (1 μM) for 24 or 48 h. Cells were stained with SA-β-gal. Boxes indicate magnified regions displayed in the right panel. *P < 0.05, **P < 0.03. **d** Nutlin-3-induced growth suppression is overcome by Si-Lamin A. HCT p53+/− cells were transfected with Si-Lamin A (2.5 μg/ml, 48 h) following Nutlin-3 treatment (1 μM, 1–3 Day). **e** Lamin A/C interacts with polycomb complex components, MEL-18, and BMI-1. Immunoprecipitation (IP) assay showed exogenous binding of GFP-tagged Lamin A and C (GFP-Lamin A, GFP-Lamin C) with FLAG-tagged MEL-18 and BMI-1 (FLAG-MEL-18, FLAG-BMI-1). **f** BMI-1 binds with the middle region of Lamin A. FLAG-tagged BMI-1 lysates were mixed with His-tagged Lamin fragment recombinant proteins (His-Lamin A N-T, His-Lamin A M) followed by IP assay. BMI-1 especially binds to Lamin N-terminal region. **g** Scheme of binding region of p53, Lamin A, and MEL-18/BMI-1. **h** RT-PCR shows decreased expression of p16 by overexpression of MEL-18 and BMI-1. Senescence markers p16 and DcR2 are downregulated by transfected overexpression of MEL-18 and BMI-1. HEK293 cells were transfected with FLAG-tagged MEL-18 and BMI-1 (1.5 μg/ml, 48 h). After transfection, RT-PCR was performed. **i** Western blotting analyses showing decrease of BMI-1 by overexpression of Lamin A and progerin. HCT p53+/− cells were transfected GFP-tagged Lamin A and Proerin (1.5 μg/ml, 48 h). After transfection, cell extracts were analyzed by western blotting using specific antibodies. Actin is used as the loading control. **j** Abrogation of MEL-18- and BMI-1-mediated p16 downregulation by Lamin A overexpression. Luciferase activities of p16 are decreased by FLAG-tagged MEL-18 and BMI-1 overexpression (1.5 μg/ml, 48 h), which are recovered by Lamin A co-transfection (2.5 μg/ml, 48 h). *P < 0.05, **P < 0.03, ***P = 0.001, NS, not significant

Although we presented that p53 stabilized Lamin A/C expression via direct binding, the mechanism of how p53 elevated p16/INK4A expression was still unclear. To reveal this, we checked the involvement of Lamin A/C in p16/INK4A expression. Induction of p16/INK4A promotor activity was obviously abolished by elimination of Lamin A/C (Fig. 6a). Similarly, elevated p16/INK4A promotor activity by Nut treatment was completely blocked by Si-Lamin A (Fig. 6b). In addition, elimination of Lamin A inhibited Nut-induced SA-β-gal expression (Fig. 6c) and growth suppression (Fig. 6d). Similar results were obtained from Etop-induced senescence. Si-Lamin A inhibited SA-β-gal expression (Supplementary Fig. 12A) and growth suppression (Supplementary Fig. 12B). However, p53−/− cells was not affected by Si-Lamin A (Supplementary Fig. 12C). To confirm that Lamin A was critical for senescence, we transfected Lamin A into HCT p53−/− cells and checked senescence markers. Overexpression of Lamin A induced p16/INK4A (Supplementary Figs. 12D and 12E) but suppressed H3K9me3 (Supplementary Figs. 12F and 12G). SA-β-gal expression was also induced by overexpression of Lamin A as strongly as p53 transfection (Supplementary Fig. 12H). These results strongly suggest that elevated Lamin A expression is enough to induce p16/INK4A expression and senescence.

### Lamin A inhibits BMI-1, a transcriptional repressor of p16/INK4A

To address the mechanism involved in Lamin A-mediated p16/INK4A induction, we searched previous literatures and found that Lamin A/C could bind to BMI-1 and MEL-18^38,39^ known to be PRC1 proteins^37^ and transcriptional repressors of p16/INK4A^40,41^. Immunoprecipitation (IP) analysis showed that Lamin A/C could interact with BMI-1 and MEL-18 (Fig. 6e). The binding affinity was observed for the middle region, not the C-terminal region, of Lamin A. It was not conserved in PRG or Lamin C (Fig. 6f and Supplementary Figs. 13A and 13B). IP analysis also showed similar binding affinity of Lamin A and PRG to BMI-1 and MEL-18 (Supplementary Fig. 13C and 13D). Hence, we conclude that the middle region of Lamin A is responsible for its binding with BMI-1 and MEL-18, whereas p53 binding is achieved at the N-terminal region of Lamin A/C (Fig. 6g). Consistently with previous reports^40,42^, BMI-1 and MEL-18 suppressed p16/INK4A expression at transcription level (Fig. 6h). Overexpression of Lamin A or PRG suppressed the expression of BMI-1 (Fig. 6i and Supplementary Fig. 13E) and MEL-18 (Supplementary Fig. 13E). Repression of p16/INK4A promotor activity by transfection of BMI-1 or MEL-18 was recovered by Lamin A co-transfection (Fig. 6j).

### p53/Lamin A/C promotes BMI-1/MEL-18 degradation

**Fig. 7:**
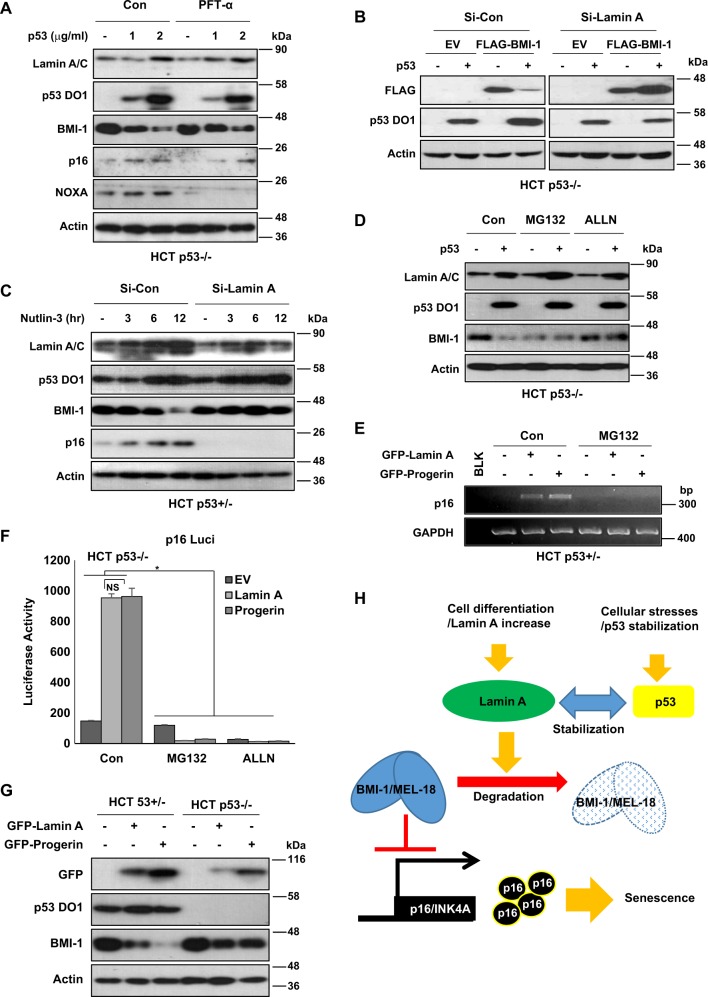
Lamin A/p53 suppresses MEL-18 and BMI-1. **a** Western blotting analyses showing p53-dependent BMI-1 decrease. Varying doses of p53 suppresses BMI-1 expression in HCT p53−/− cells. Dose dependency of p53 (1, 2 μg/ml, 48 h) vectors were transfected and PFT-α (20 μM, 24 h) were treated. Western blotting analysis was performed with specific antibodies. Western blotting data of three independent experiment are shown. **b** BMI-1 suppression of Lamin A-mediated p53. Suppression of FLAG-tagged BMI-1 (FLAG-BMI-1) is abrogated by Si-Lamin A. Exogenous BMI-1 (1.5 μg/ml, 48 h) was co-transfected with Si-Con or Si-Lamin A (2.5 μg/ml, 48 h) and cell extracts were subjected to western blotting analysis. Actin is the loading control. **c** Western blotting depicts BMI-1 suppression by p53 stabilization and its abrogation by Si-Lamin A. Stabilization of p53 by Nutlin-3 treatment (1 μM, 3–12 h) downregulates BMI-1 expression, whereas Si-Lamin A blocks the suppression. **d** Suppression of BMI-1 by p53 was recovered by inhibition of proteasome degradation. p53 suppresses BMI-1, whereas proteasome inhibitor MG132 and ALLN block BMI-1 degradation. HCT p53−/− cells were transfected with p53 vector (1.5 μg/ml, 48 h). After transfection, cells were treated with MG132 (10 μM) and ALLN (5 μg/ml) for 12 h. Cell extracts were subjected to western blotting using specific antibodies. **e** Inhibition of proteasome degradation of BMI-1 suppresses p16 expression. RT-PCR images showing that GFP-tagged Lamin A and progerin (GFP-Lamin A, GFP-Progerin)-induced p16 expression, whereas such induction was blocked by MG132 treatment. HCT p53+/− cells were transfected with GFP-Lamin A or Progerin (1.5 μg/ml, 48 h) and treated with MG132 (10 μM, 12 h). After treatment, cells were subjected to RT-PCR. **f** p16 transcription is abolished by proteasomal inhibition. p53-negative HCT p53−/− cells showed elevated p16 luciferase activity by Lamin A/Progerin transfection. Lamin A/Progerin-induced p16 luciferase activity was abolished by MG132 and ALLN treatment. p16-Luciferase vector (1.5 μg/ml) was transfected with GFP-tagged Lamin A or Progerin (1.5 μg/ml) for 48 h following treatment with MG132 (10 μM, 12 h) and ALLN (5 μg/ml, 12 h). After treatment, cells were subjected to luciferase assay. *P < 0.05; NS, not significant. **g** Lamin A/Progerin suppresses BMI-1 differently in p53 isogenic HCT cells. Suppression of BMI-1 expression by GFP-tagged Lamin A and progerin (GFP-Lamin A, GFP-Progerin) is more obvious in p53-positive HCT p53+/− cells than that in HCT−/− cells. In addition, progerin-induced suppression of BMI-1 is more dramatic compared with Lamin A-induced BMI-1 reduction in HCT p53+/− cells. p53 isogenic HCT cells were transfected with GFP-Lamin A or Progerin (1.5 μg/ml, 48 h). After transfection, cell extracts were analyzed with western blotting. **h** Model illustrating Lamin A-mediated p53 induces senescence. p53 stabilization and its elevated expression increase Lamin A. In Lamin A-mediated mechanism, p53 induces proteasome degradation of polycomb complex component MEL-18/BMI-1. MEL-18/BMI-1 degradation leads to p16 transcriptional activation, which results in cellular senescence

As we revealed that Lamin A/C (including PRG) could suppress BMI-1/MEL-18 expression, we next checked the effect of p53 on BMI-1/MEL-18 expression. Transfection of p53 into p53-null cells (Fig. 7a) or increased endogenous p53 expression in HCT116 cells (Supplementary Fig. 14A) suppressed the expression of BMI-1. Immunofluorescence staining also showed reduction of BMI-1 and MEL-18 by p53 transfection (Supplementary Fig. 14B). As reduction of BMI-1 was not abolished by PFT-α, the induction of p16/INK4A was also detected in PFT-α-treated cells (Fig. 7a and Supplementary Fig. 14A). BMI-1 reduction and increased expression of p16/INK4A were also detected in HCT p21−/− (Supplementary Fig. 14C), HCT Bax−/− (Supplementary Fig. 14D), and HCT Puma−/− (Supplementary Fig. 14E) cell lines after Nut treatment. UV-induced p53 activation suppressed BMI-1 expression but induced p16/INK4A in HCT116 isogenic cell lines (Supplementary Figures 14F-H). These results indicate that major p53 downstream target genes are not associated with BMI-1 reduction or p16/INK4 increase in Nut-treated or physiologically DNA-damage-induced condition.

p53 also suppressed exogenous BMI-1 and MEL-18 expression in HCT116, PC3, and H1299 p53-null human lung cancer cell lines (Supplementary Figs. 15A and B). However, elimination of Lamin A completely blocked p53-mediated BMI-1 reduction (Fig. 7b and Supplementary Fig. 15C). The reduction of BMI-1 by Nut treatment (Fig. 7c) or transfection (Supplementary Fig. 15D) was completely blocked by Lamin A knockdown. Under this condition, p16/INK4A induction was also diminished by Si-Lamin A (Fig. 7c and Supplementary Fig. 15D). Si-Lamin A blocked transcriptional induction of p16/INK4A in response to DNA damage (Supplementary Fig. 15E) and Nut treatment (Supplementary Fig. 15F). Si-Lamin A also blocked p16/INK4A transcription under p53 overexpression or stabilization condition regardless of PFT-α treatment (Supplementary Figs. 15G and 15H). Considering that exogenous BMI-1 and MEL-18 were eliminated by p53 or Lamin A/C, we thought their regulation might be achieved at the posttranslational level. Hence, we first tested proteasome-mediated degradation. Proteasome inhibitors MG132 and ALLN blocked p53-induced (Fig. 7d and Supplementary Fig. 16A) and Lamin A/PRG-induced BMI-1 reduction (Supplementary Fig. 16B). MG132 blocked Lamin A/PRG and p53-mediated p6/INK4A induction (Fig. 7e and Supplementary Fig. 16C). Induction of p16/INK4A promotor activity by Lamin A or PRG was also abolished by proteasome inhibitors MG132 and ALLN (Fig. 7f and Supplementary Fig. 16D). Moreover, PRG-induced BMI-1 reduction (Fig. 7g and Supplementary Fig. 16E and F) was more obvious in HCT116 parental cell line. In contrast, Lamin A or PRG did not show dramatic difference in BMI-1 reduction. This result might be related to additional binding of p53 and PRG.

## Discussion

It is well known that p53 and its isoforms are very important factors of cellular senescence^8–11^. However, detailed molecular mechanism about p53-induced senescence has not been clearly demonstrated yet. Despite obvious relevance between p16/INK4A and senescence^22,23^, how p16/INK4A is elevated under senescence condition is unclear. In this study, we investigated p53-induced senescence and p16/INK4A induction. Our results showed that when p53 was increased by various stimulations, Lamin A/C was stabilized (Fig. 7h). As Lamin A/C was not expressed in stem cells but increased in differentiated cells^6,7^, we speculated that Lamin A/C induction could be an important trigger for senescence. PRG, an abnormal splicing product of Lamin A/C, is known to be a causal factor of HGPS, a premature aging disease. Previous results have shown that PRG can inhibit Lamin A/C dissemination and induce nuclear deformation^4^. Abnormal nuclear shape mainly accompanies cellular senescence. Such nuclear deformation occurs from Lamin A abnormalities (localize interruption, immature expression, deregulated expression, etc.), but not from Lamin B abnormalities^43^. In this study, we observed that p53-mediated Lamin A/C increase promoted nuclear deformation (very similar phenotype of HGPS cells). Considering that PFT-α (inhibitor of p53 transcription activity)-treated cells (Fig. 3) and p53 target gene-deleted cells (Supplementary Fig. 4) showed response similar to untreated and parental cells for p53-mediated Lamin A/C and p16/INK4 induction, we deduced that the transcriptional activity of p53 might be dispensable for p53-induced senescence. Instead, characteristics of p53-induced senescence resembled HGPS. Indeed, loss of p53 is known to partially suppress premature aging phenotype in HGPS model^44^.

In this study, we also provided a novel p16/INK4A regulation mechanism involving elimination of PRC1 (BMI-1 and MEL-18). Although we did not observe reduction of endogenous MEL-18, BMI-1 expression was obviously repressed by p53 in addition to expression of Lamin A/C and PRG. In particular, PRG showed apparent effect on BMI-1 repression (Figs 6g, 7g and Supplementary Figs. 16B, 16E). Concerning this, we speculated that additional binding of p53 and PRG (Fig. 5g) would be related to the strong effect of PRG on BMI-1 reduction and senescence. To get more clear evidence for this, further study is needed.

This study provides several evidences for the involvement of Lamin A/C in p16/INK4A induction. Elimination of Lamin A through mall interference RNA (Si-RNA) completely blocked p53-mediated p16/INK4A induction (Fig. 6a, b) and BMI-1 reduction (Fig. 7c and Supplementary Fig. 15D). Thus, we postulate that, in embryonic stem cells that do not express Lamin A/C, p53-mediated senescence might be impaired and another pathway might be involved in stem senescence.

Taken together, we propose a new p53-mediated cellular senescence pathway. Activated p53 stabilizes Lamin A/C and induces HGPS-like nuclear deformation. Increased Lamin A/C then promotes the degradation of BMI-1 that blocks p16/INK4A expression. Thus, p53 activation can induce p16/INK4A-mediated cellular senescence (Fig. 7h). Our results provide a new insight to the understanding p53-mediated senescence regarding why and how differentiated cells enter senescence.

## Materials and methods

### Cell culture and reagents

HCT116 (p53+/−) cells and its isogenic cell lines (p53−/−, p21−/−, PUMA−/−, and Bax−/−) were provided by Dr. B. Vogelstein (Johns Hopkins University). Human cell lines used in this study were obtained from the American Type Culture Collection (Manassas, VA, USA) and the Korean Cell Line Bank (Seoul, South Korea). Cell lines were maintained in liquid media (RPMI-1640 or Dulbecco’s modifed Eagle’s medium) supplemented with 10% fetal bovine serum (FBS) and 1% penicillin–streptomycin at 37 °C with 5% CO_2_. Human fibroblast cells (9-year-old female) were obtained from Coriell Cell Repositories (Camden, NJ, USA) and maintained in Eagle's minimal essential medium supplemented with 15% FBS, 2 mM glutamine, and 26 mM HEPES without antibiotics. Nutlin-3 (N6287) was purchased from Sigma. PFT-α (506134), MG132 (474790), ALLN (208719), and Etoposide (341205) were obtained from Calbiochem (Darmstadt, Germany). Doxorubicin hydrochloride (Adr; 2252) was purchased from Tocris Bioscience (Bristol, UK).

### Recombinant proteins

To produce recombinant proteins, human Lamin A N-terminal domain fragment (His-Lamin A N; residue 1–300) and Lamin A M region fragment (His-Lamin M; residue 301–564) were cloned into His-tagged pPROEXHT. These recombinant proteins were purified using a nickel column. Recombinant Lamin A C-terminal region (Lamin A–C) and PRG C-terminal region (Progerin C) were produced by cloning 100 AA from upstream of the termination codon through PCR. p53 fragments (1–93 and 93–292) were expressed in *Escherichia coli* as GST-fusion proteins. Each fragment was loaded onto glutathione (GSH)-agarose and then eluted with a buffer containing 20 mM reduced GSH after extensive washing. Eluted fractions were further purified using an anion-exchange chromatography (HitrapQ).

### Immunoblotting

Protein extraction from cells was accomplished using RIPA buffer (50 mM Tris-Cl, pH 7.5, 150 mM NaCl, 1% NP-40, 0.1% SDS, and 10% sodium deoxycholate). Samples were separated on SDS-polyacrylamide gel electrophoresis (PAGE) and transferred to polyvinylidene difluoride membrane. Blotted membranes were blocked with 3% skim milk for 1 h followed by incubation with specific primary antibodies. The following antibodies were used in this study: Lamin A/C (sc-376248), GST (sc-138), green fluorescent protein (GFP) (sc-9996), p53 DO1 (sc-126), Actin (sc-47778), His (sc-8036), nuclear factor-κB (sc-372), IκBα (sc-371), and p21 (sc-397) from Santa Cruz Biotechnology (Santa Cruz, CA, USA); α-FLAG (F3165) from Sigma (St. Louis, MO, USA); Emerin from Novocastra (New castle, UK); PUMA (4976), phospho-Erk (9101), total-Erk (9102), phospho(S15)-p53 (9286), phospho(S20)-p53 (9287), and BMI-1(5856) from Cell Signaling Technology (Danvers, MA, USA); NOXA (ALX 804-408-C100) from Enzo Life Sciences (Farmingdale, NY, USA); p16 (10883-1-AP) from Proteintech (Rosemont, IL, USA); and Histone H3k9me3 (ab8898) from Abcam (Cambridge, UK). Horseradish peroxidase-conjugated goat anti-mouse, goat anti-rabbit, and mouse anti-goat IgG antibodies (Pierce, Thermo Fisher Scientific, Inc., Rockford, IL, USA) were used as secondary antibodies.

### Protein–protein interaction analyses

To analyze protein–protein interaction, GST-pulldown assay and IP experiments were performed. To detect the interaction, GST-bead-fused p53 N-terminal region (GST-p53 N-T) or middle region (GST-p53 M) was incubated with HEK293 cell lysate transfected with either GFP-tagged Lamin A (GFP-Lamin A), Lamin B (GFP-Lamin B), Lamin C (GFP-Lamin C), or PRG (GFP-PRG) at room temperature (RT) for 3 h. After washing once each with phosphate-buffered saline (PBS) and RIPA buffer, precipitates were collected and subjected to SDS-PAGE and western blotting using anti-GFP and GST. For IP assay, whole-cell lysates expressing FLAG-tagged MEL-18, BMI-1, and GFP-tagged Lamin A/C were incubated with anti-FLAG antibody at 4 °C for 2 h, followed by incubation with protein A/G agarose beads (Invitrogen, Carlsbad, CA, USA) at 4 °C for 2 h. After centrifugation and washing with RIPA buffer, immunocomplexes were separated by SDS-PAGE and subjected to western blotting with anti-FLAG, Actin, and GFP. For endogeneous IP assay, whole-cell lysates were incubated with designated antibody (anti-Lamin A/C, anti-p53 DO1) at 4 °C for 4 h followed by incubation with protein A/C agarose beads at 4 °C for 2 h. Binding of His-tagged Lamin A N-terminal or M region with FLAG-tagged BMI-1 was performed by incubating FLAG-tagged BMI-1 lysates with each His-tagged Lamin A N-terminal and M region recombinant protein at 4 °C for 2 h followed by incubation with anti-His antibody at 4 °C for 2 h and then with protein A/G agarose beads at 4 °C for 2 h. After centrifugation and washing with RIPA buffer, these immunocomplexes were separated by SDS-PAGE and subjected to western blotting with designated antibodies.

### Immunofluorescence staining

Cells were cultured on coverslips, washed with PBS, fixed with 4% parafprmaldehydefor 30 min at RT, and then permeabilized in 0.1% Triton X-100/PBS at RT for 10 min. After treatment with blocking solution (anti-Human antibody diluted 1:500 in PBS) for 1 h, cells were incubated with anti-Lamin A/C (1:500), p16 (1:300), and H3K9me3 (1:300) in blocking solution overnight at 4 °C. Finally, cells were incubated with fluorescein isothiocyanate (FITC) and Rhodamine-conjugated secondary antibodies at 4 °C for 6 h. The nucleus was stained with DAPI (4, 6-diamidino-2-phenylindole) for 10 min. After cells were washed three times with PBS, coverslips were mounted with mounting solution (H-5501; Vector Laboratories (Burlingame, CA, USA) and analyzed by fluorescence microscopy (Zeiss, Axioplan 2) at × 400 magnification.

### Transfection of vectors and Si-RNA

GFP-fused PRG (GFP-PRG) and GFP-fused Lamin A (GFP-Lamin A) expression vectors were kindly provided by Misteli T. (National Cancer Institute). GFP-fused Lamin B (GFP-Lamin B) and GFP-fused Lamin C (GFP-Lamin C) expression vectors were generously provided by Lammerding J. (Brigham and Women’s Hospital, Harvard Medical School). p53 vector was a kind gift from Dr Kim S. (Seoul National University). SV40 Large T (LT) antigen vector was purchased from Addgene (Massachusetts, USA^36^). Sh-p53 vectors were provided by Mayo L.D. (Herman B. Wells Center for Pediatric Research/Indiana University School of Medicine). FLAG-fused MEL-18 and BMI-1 vectors were provided by Dr Maertens G.N. (London Research institute). Jet-pei (Polyplus Transfection, New York, USA) was used in transfection for mammalian expression of these vectors. Briefly, vector (1.5 µg) was mixed with 1.5 µl of Jet-pei reagent in 150 mM NaCl solution. The mixture was incubated at RT for 15 min. After incubation, the mixture was added to cells. After 3 h of incubation, serum-free medium was replaced with culture medium supplemented with 10% FBS.

For in vitro gene knockdown, Si-RNA against target proteins were generated (Cosmo Genetech, Seoul, Korea). Target sequences of Si-RNA were as follows: Si-Con (5′-AAT TCT CCG AAC GTG TCT CGT TTC AAC CTT ACG AGA CAC GTT CGG AGA ATT-3′), Si-Lamin A (5′-TGA CCT GAA CCT CTT CTT GTA GTT CAA GAG ACT ACA AGA AGA CCT TCA GGT CA-3′), and Si-p53 (5′-GCA TCT TAT CCG AGT GGA ATT CAA GAG ATT CCA CTC GGA TAA GAT GC-3′). Transfection was performed for 48 h using Jet-pei (Polyplus Transfection, New York, USA) reagent according to the manufacturer’s protocol. In brief, cells seeded on the previous day were washed with PBS and incubated with DNA/Jet-pei mixture for 4 h under serum-free condition.

### RNA isolation and RT-PCR

For RT-PCR, total cellular RNA was extracted using RNA extraction kit (Qiagen, Maryland, USA). After measurement of RNA concentration, 1 µg of total RNA was reverse transcribed to cDNA using MMLV RT (Moloney murine leukemia virus reverse transcriptase: Invitrogen, CA, USA) and random hexamer. RT-PCR was performed with specific primers of target genes as follows: p16 (5′-CAA CGC ACC GAA TAG TTA CG-3′ and 5′-ATC TAT GCG GGC ATG GTT AC-3′), Lamin A (5′-AAG GAG ATG ACC TGC TCC ATC-3′ and 5′-TTT CTT TGG CTT CAA GCC CCC-3′), p21 (5′-GAG CGA TGG AAC TTC GAC TT-3′ and 5′-CAG GTC CAC ATG GRC TTC CT-3′), Bax (5′-GCT TCA GGG GTG AGT TTG AGG-3′ and 5′-CGG AAT GTT TGC GCT GAG TTG-3′), Puma (5′-GTC CTC AGC CCT CGC TCT-3′ and 5′-CTG CTG CTC CTC TTG TCT CC-3′), Noxa (5′-AAG ATT ACC GCT GGC CTA CTG-3′ and 5′-GTC TAC TGA TTT ACT GGC CCC-3′), and GAPDH (5′-ATC TTC CAG GAG CGA GAT CCC-3′ and 5′-AGT GAG CTT CCC GTT CAG CTC-3′).

### Measurement of cell viability

To examine cell viability, cells were incubated with 0.5 mg/ml of MTT (3-(4,5-dimethylthiazol-2-yl)-2,5-diphenyltetrazolium bromide) solution (475989; Merck, Darmstadt, Germany) at 37 °C for 4 h. After removing excess solution and washing with PBS, precipitated materials were dissolved in 200 µl dimethyl sulfoxide and quantified by measuring absorbance at 540 nm.

### Colony-forming assay

To determine cell proliferation capability, cells were seeded into 35 × 10 mm culture dishes at a low density (~2,000 cells/well). After designated transfection or chemical treatment, media were removed and wells were washed with PBS. These dishes were stained with crystal violet and observed microscopically.

### Flow cytometry and cell death assay

For cell cycle and cell death assay, cultured cells were detached, collected, and washed with PBS. After discarding the supernatant, cells were washed with PBS and fixed with 70% ethanol for 1 h. Fixed cells were washed with PBS and then incubated with 10 μg/ml propidium iodide (PI) and 100 μg/ml RNase A. For cell death assay, Annexin V-FITC Apoptosis Detection Kit (BioTool, TX, USA) was used following the manufacturer’s protocol. Briefly, cells were detached and collected. Collected cells were suspended in 1 × binding buffer for 10 min at RT. After incubation, cells were stained with PI staining solution and Annexin V-FITC mixed with 1 × binding buffer. Flow cytometry analysis was performed using FACSCANTO II (BD Biosciences, San Jose, CA, USA).

### SA-β-gal activity assay

For SA-β-gal activity staining, cells were washed once with PBS (pH 7.2) and fixed with PBS containing 0.5% glutaraldehyde. After fixing, cells were washed with PBS and stained with X-gal solution (Cell Signaling Technology, Danvers, MA, USA) at 37 °C overnight.

### Luciferase assay

To estimate transcriptional activity of p16, cells were co-transfected with p16 luciferase vectors and plasmid vectors (p53, Lamin A) or Si-RNA (Si-Con, Si-Lamin A) for 48 h. After that, cells were treated with Nut for designated time. After washing with PBS, cells were lysed with lysis buffer (Promega, Madison, WI, USA). Luciferase activity was determined with a luminometer (MicroDigital, Gyeonggi-do, South Korea).

### Cell counting

For cell growth arrest experiment, cells were seeded into six-well plates at a low density (~ 5 × 10^5^cells/well). After designated chemical treatment, cells were collected with media and stained with Trypan blue (GIBCO, Grand Island, NY, USA) at RT for 5 min. The number of viable cells (unstained) was counted using a hemocytometer.

### Nuclear deformation counting

For nuclear deformation cell counting, immunofluorescence images with Lamin A/C antibody were used. Depending on Lamin A staining, nuclear membrane showing abnormalities was counted in randomly selected fields and expressed as percentage of total cells counted. Nuclear membrane abnormalities were determined based on the following: Lamin A/C lining was (1) extruded or engulfed, (2) was at least one bleb, and (3) irregular contour. Counting of cells with nuclear deformation was performed by three independent observers who were blinded to transfection and chemical treatment group. For histone H3K9me3 intensity, images were quantified through the “color histogram” function of the Image J software (National Institute of Health, NIH). Fluorescence intensities were subtracted with background signals.

### UV-irradiation

UV cross-linker (UV 254 nm) model CL-1000 (Ultra-Violet Product Ltd, Cambridge, UK) was used to irradiate cells. Plates containing seeded cells were exposed to a dose of 70 mJ/cm^2^. After irradiation, plates were incubated under standard cell culture conditions for indicated time periods (3 h and 6 h).

### Statistical analysis

Student’s *t*-test was used for comparisons of two groups. *P*-value < 0.05 was considered significant. Error bars indicate SD. Data for all figures are expressed as means ± SD of at least three independent experiments.

## Supplementary information

Supplementary Figure legend

Supplementary Figures
