# Supplementary material for: p53 induces senescence through Lamin A/C stabilization-mediated nuclear deformation
Source: Cell Death Dis. 2019 Feb 6;10(2):107. doi: 10.1038/s41419-019-1378-7 (PMC6365587; doi:10.1038/s41419-019-1378-7)
Supplement: Supplementary file 1 — Supplementary Figure legend [file 41419_2019_1378_MOESM1_ESM.docx]

**Supplementary Figure Legends**

**Supplementary Figure 1.** p53 induces nuclear deformation. Immunofluorescence (IF) staining showed that nuclear deformation was increased in p53 null HCT116 cells (HCT116 p53-/-) by p53 transfection in a dose-dependent manner. HCT p53-/- cells were transfected with p53 at different doses (1-5 μg/ml) for 48 hr. After transfection, cells were IF stained with p53 (Green), Lamin A (Red), and counterstained with DAPI (Blue).

**Supplementary Figure 2.** Nutlin-3 induced nuclear deformation and cellular senescence are dependent on p53 stabilization. **(A)** p53 negative cells have no effect on nuclear deformation. Nutlin-3 induced p53 stabilization did not show nuclear deformation in HCT p53-/- cells. As a positive control, only p53 transfection affected nuclear deformation. HCT p53-/- cells were transfected with p53 (1.5 μg/ml, 48 hr) or treated with Nutlin-3 (1 μM) for different time periods (24, 48 hr). After transfection or treatment, cells were immunofluorescence stained with Lamin A/C (Red) and counter stained with DAPI (Blue). **(B)** Cells with nuclear deformation in (A) are calculated. *P < 0.01, NS, not significant. **(C)** Western blot analyses showing unchanged expression of endogenous Lamin A/C in HCT p53-/- cells after Nutlin-3 treatment. As a positive control, only p53 transfection increased the expression of Lamin A/C and p16. HCT p53-/- cells were transfected with p53 (1.5 μg/ml, 48 hr) or treated with Nutlin-3 (1 μM) for different time periods (24, 48 hr). After transfection or treatment, cell extracts were analyzed by western blot with specific antibodies. **(D)** Nultin-3 did not induce cellular senescence in p53 null cells. SA-β-gal staining showed that p53 null HCT116 cells were not affected by Nutlin-3 induced senescence. As a positive control, only p53 transfection affected cellular senescence (left). Results of counting of β-gal positive cells (right) are shown. HCT p53-/- cells were transfected with p53 (1.5 μg/ml, 48 hr) or treated with Nutlin-3 (1 μM) for different time periods (24 and 48 hr). After transfection or treatment, cells were subjected to SA-β-gal staining. Boxes indicate magnified regions displayed in the right panel. *P < 0.01, NS, not significant.

**Supplementary Figure 3.** p53 stabilization induces growth arrest. **(A)** Nutlin-3 induced growth arrest in p53 wild-type cells. Cell counting showed that Nutlin-3 induced cell growth arrest only in p53 wild-type HCT p53+/- (middle) and A549 cells (right), but not in p53 null HCT p53-/- cells (left). Cells were treated with Nutlin-3 (1 μM) for different days (1-5 Days). Cell counting was performed using a hemocytometer. *****P < 0.01, *****P < 0.001, NS, not significant. **(B)** Proliferative signals were not affected by Nutlin-3 treatment. IF staining showed that proliferative marker Ki-67 was not affected by Nutlin-3 (1 μM and 24, 48hr) (left). Results of counting of Ki-67 positive cells (middle) and signal intensity (right) based on immunofluorescence staining data are shown. NS, not significant.

**Supplementary Figure 4.** p53 stabilization shows cellular senescence phenotypes. **(A)** Nutlin-3 induced cell enlargement based on immunofluorescence staining of cellular fiber (Phalloidin; Red) and focal adhesion molecule (Paxillin; Green). Staining showed that Nutlin-3 induced cell enlargement and co-staining of Phalloidin with Paxillin in p53 wild-type HCT116 cell. HCT p53+/- cells were treated with Nutlin-3 (1 μM) for different time periods (24 and 48 hr). After treatment, cells were IF stained with Phalloidin (Red), Paxillin (Green), and counterstained with DAPI (Blue). Arrow heads indicate co-localization of Phalloidin and Raxillin. **(B)** Paxillin expression was increased by Nutlin-3 treatment. Western blot images showed increase of Paxillin and p16 by Nutlin-3 treatment in a time-dependent manner. HCT p53+/- cells were treated with Nutlin-3 (1 μM) for different time periods (24 and 48 hr). After treatment, cell extracts were subjected to western blot. **(C)** Counting of single cell size increase (left) and number of enlargement cell (right) based on the immunofluorescence staining in (A). *****P < 0.001. **(D)** Induced cell size by Nutlin-3 did not occur in p53 null cells. IF staining showed no change of cell enlargement or co-staining of Phalloiding/Paxillin in HCT p53-/- cells by Nutlin-3. HCT p53-/- cells were treated with Nutlin-3 (1 μM) for different time periods (24 and 48 hr) followed by IF staining with Phalloidin (Red), Paxillin (Green), and counterstaining with DAPI (Blue). **(E)** Paxillin expression was not increased by Nutlin-3 in p53 null cells. HCT p53-/- cells were treated with Nutlin-3 (1 μM) for different time periods (24 and 48 hr). After treatment, cell extracts were subjected to western blot. **(F)** Counting of single cell size increase (left) and number of enlargement cell (right) based on immunofluorescence staining in (D). NS, not significant.

**Supplementary Figure 5.** p53 stabilization induced cellular senescence is not related to cell death. **(A)** Nutlin-3 treatment did not trigger cell death. Treatment with Nutlin-3 at different doses (1-8 μM, 48 hr) or time periods (1 μM, 3-48 hr) did not affect p53 isotype in HCT116 cells. NS, not significant. **(B)** Nutlin-3 treatment did not lead to cell death. FACS images show no death signal after treatment with Nutlin-3 (1 μM) for different time periods (24 and 48 hr). Co-treatment with PFT-α (20 μM) did not lead to cell death either. Values are means ± SEM (n = 5 per group). **(C)** Nultin-3 treatment did not lead to cell apoptosis. Annexin V staining after FACS analysis showed no relationship between Nultin-3 treatment (1 μM) for different time peroids (24, 48 hr) and cell apoptosis. Adriamycin treatment (2 μg/ml, 48 hr) was used as apoptotic cell control. Values are means ± SEM (n = 5 per group). **(D)** Nutlin-3 treatment group did not show cell death signal in p53 null cells. FACS analysis data showed that even p53 null HCT116 cells did not show cell death after Nutlin-3 (1 μM) treatment for different time periods (24, 48 hr). Values are means ± SEM (n = 6 per group). Data of three independent experiments (B-D) are shown.

**Supplementary Figure 6.** UV irradiated p53 stabilization induces nuclear deformation and increases Lamin A/C and p16 expression. **(A)** Proteasomal degradation inhibition is not related to p53 mediated nuclear deformation and increase of Lamin A/C expression. Immunofluorescence images revealed that proteasomal degradation inhibitor MG132 had no effect on nuclear deformation. HCT p53+/- cells were treated with MG132 (10 μM, 12 hr) followed by IF staining with Lamin A/C (Red) and counterstained with DAPI (Blue). **(B)** Western blot analyses show increased p53 expression levels by MG132. Lamin A/C level remained unchanged in a p53 expression dependent manner. HCT p53+/- cells were treated with MG132 (10 μM, 12 hr). After treatment, cell extracts were subjected to western blot. **(C)** Nuclear deformation is induced under UV irradiation. IF images showing cells with nuclear deformation in HCT p53+/- cells in a time dependent manner under conditions of UV irradiation (70 J/m^2^). HCT p53+/- cells were exposed to UV (70 J/m^2^ and 3, 6 hr). After exposure, cells were IF stained with Lamin A/C (Red) and counter stained with DAPI (Blue). **(D)** Cells with nuclear deformation in (C) are calculated. *P < 0.03. **(E)** Lamin A/C expression is upregulated by UV irradiation. Western blot analyses showing increased Lamin A/C expression through UV induced p53 increase. **(F)** Cells with nuclear deformation in (G) are calculated. NS, not significant. **(G)** Nuclear deformation is not induced in p53 negative cells by UV irradiation. Nuclear deformation is not detected in HCT p53-/- cells after UV irradiate condition (70 J/m^2^) for different time periods. HCT p53-/- cells were exposed to UV (70 J/m^2^) for 3 and 6 hr. After exposure, cells were IF stained with Lamin A/C (Red) and counter stained with DAPI (Blue).

**Supplementary Figure 7.** Inhibition of p53 transcriptional activation has no effect on p53-induced p16 expression. **(A-C)** p53 overexpression (A), stabilization (B), and DNA-damage stimuli induction (C) increase p16/INK4A at transcriptional level irrespective of treatment with PFT-α. PFT-α only affected transcriptional levels of p53 target genes. (A) HCT p53-/- cells were transfected with p53 vector (1-3 μg/ml, 48 hr) with or without treatment with PFT-α (20 μM, 24 hr). (B) HCT p53+/- cells were treated with Nutlin-3 (1 μM) for different time periods (3, 6, and 12 hr) with or without PFT-α (20 μM). (C) HCT p53+/- cells were treated with Adriamycin (2 μg/ml) for different time periods (3, 6, and 12 hr) with or without treatment with PFT-α (20 μM). After transfection or chemical treatment, RT-PCR was performed. **(D)** Transcription inhibition of p53 target gene by PFT-α. Using luciferase activity analysis, p53 target gene (GADD45-Luci) was found to be transcriptionally inhibited by PFT-α (20 μM, 24 hr), but not in p16-Luci. *****P < 0.01, **P<0.001, NS, not significant.

**Supplementary Figure 8.** Transcription activity of p53 is dispensable for nuclear deformation. **(A, C)** Cells with nuclear deformation by Nutlin-3 treatment with or without treatment by PFT-α. Nuclear deformation in p53 wild type A549 (A) and MCF-7 (C) cells after treatment with Nutlin-3 (1 μM) for different time periods (24 and 48 hr). PFT-α co-treatment (20 μM) did not affect Nutlin-3 induced nuclear deformation. **(B)** Nuclear deformation rates in (A) were calculated. *P<0.03, NS, not significant. **(D)** Nuclear deformation rates in (C) were calculated. *P<0.03, NS, not significant. **(E)** Nuclear deformation by Etoposide (Etop) treatment with or without PFT-α. Treatment with Etop (10 μM) for different time periods (24 and 48 hr) induced nuclear deformation in HCT p53+/- cells. PFT-α did not affect Etop induced nuclear deformation. **(F)** Nuclear deformation rates in (E) were calculated. *P<0.03, NS, not significant. **(G)** Adriamycin induced p53 upregulation which increased p16 transcription. HCT p53+/- cells were treated with Adr (2 μg/ml) for different time periods (3, 6, and 12 hr) with or without PFT-α (20 μM). After treatment, cells were subjected to RT-PCR.

**Supplementary Figure 9.** Wild type p53 is required for nuclear deformation. **(A, C)** Nuclear deformation induced by Nutlin-3 treatment in p53 mutant Wi-26 (A) and Capan-1 (C) cells. Cells were treated with Nutlin-3 (1 μM) for different time periods (24 and 48 hr) followed by IF staining with Lamin A/C (Red) and counterstaining with DAPI (Blue). **(B)** Cells with nuclear deformation in (A) were calculated. NS, not significant. **(D)** Cells with nuclear deformation in (B) were calculated. NS, not significant.

**Supplementary Figure 10.** Transcriptional target genes are not involved in p53-mediated senescence. **(A, C, E)** Time coursed treatment of Nutlin-3 induced nuclear deformation and decreased histone H3K9me3 in p53 target gene deficient cells. p53 target gene negative HCT cells (HCT p21-/-, HCT Bax-/-, HCT Puma-/-) showed similar effects of Nutlin-3 dependent nuclear deformation and decrease of histone H3K9me3 (right). Cells with nuclear deformation were calculated (left). Cells were treated with Nutlin-3 (1 μM) for different time periods (24 and 48 hr). After treatment, cells were immunofluorescence stained with Lamin A/C (Red), H3K9me3 (Green), and counterstained with DAPI (Blue). *P<0.01. **(B, D, F)** Western blot analyses depicting increased Lamin A/C and p16 expression in p53 target gene negative cells. p53 target negative HCT cells were treated with Nutlin-3 (1 μM) for different time periods (24 and 48 hr). After treatment, cell extracts were analyzed by western blot. LE and SE indicated long-exposure and short exposure, respectively.

**Supplementary Figure 11.** Wild type p53 stabilizes Lamin A/C. **(A)** Etoposide induced nuclear deformation. Immunofluorescence images show that even normal fibroblast (N9) treated with, physiological DNA-damage condition, Etoposide induced nuclear deformation. N9 cells were treated with Etoposide (10 μM) for different time periods (3, 6, and 12 hr). After treatment, cells were IF stained with Lamin A/C (Red), H3K9me3 (Green), and counterstained with DAPI (Blue). **(B)** p53 stabilization also leads to nuclear deformation. Immunofluorescence images of normal fibroblast cells (N9) show that p53 stabilization by Nutlin-3 induces nuclear deformation, similar to physiological stimuli. N9 cells were treated with Nutlin-3 (1 μM) for different time periods (3, 6, and 12 hr). After treatment, cells were IF stained with Lamin A/C (Red), H3K9me3 (Green), and counterstained with DAPI (Blue). **(C)** p53 upregulates Lamin A/C in a dose-dependent manner. Transfected p53 wild type (0.5-2 μg/ml, 48 hr) showed increases in expression level of Lamin A/C while emerin expression remains unaffected. **(D)** Transcriptional activity of p53 does not upregulate Lamin A or progerin. GFP tagged Lamin A or Progerin (1.5 μg/ml, 48 hr) was transfected with p53 at different doses (1-3 μg/ml, 48 hr). After transfection, cells were subjected to RT-PCR. GAPDH is used as a loading control **(E)** Decreasing Lamin A expression by p53 knock-down. Immunofluorescence images show that small hairpin RNA (Sh-RNA) of p53 decreases GFP tagged Lamin A (GFP-Lamin A) expression. MCF-7 cells were transfected with GFP-tagged Lamin A (1.5 μg/ml, 48 hr) with Sh-p53 at different doses (1, 2 μg/ml, 48 hr). After transfection, cells were IF stained with GFP (Green) and counter stained with DAPI (Blue). **(F)** Dose dependent Sh-p53 transfection shows Lamin A/C decrease. MCF-7 cells were transfected with Sh-p53 at different concentrations (5-10 μg/ml, 48 hr). After transfection, cell extracts were analyzed by western blot. Actin is used as a loading control. **(G)** Lamin A binds to middle region of p53. GST pull-down assay shows that GFP tagged Lamin A binds to p53 middle region. RKIP-GST is used as a negative control. PPT: Pellet, Sup: Supernatant. **(H, I)** Binding of p53 and Lamin A/C is weakened by SV40 large T antigen. PPT: Pellet, Sup: Supernatant. **(J)** SV40 Large T antigen affects p53 induced Lamin A and progerin expression. Two p53 wild type cells (A549 and MCF-7) show decreased expression of exogenous Lamin A and progerin by SV40 large T antigen (Large T). Cells were transfected with GFP tagged Lamin A and progerin (GFP-Lamin A, GFP-Progerin) (1.5 μg/ml, 48 hr) with or without co-transfection of SV40 Large T antigen (1.5 μg/ml, 48 hr).

**Supplementary Figure 12.** Knock-down of Lamin A recovers p53-induced cellular senescence. **(A)** Lamin A knock-down results in recovery of DNA-damage induced cellular senescence. SA-β-gal staining images show that time-dependent treatment with Etoposide induces cellular senescence which is recovered by Lamin A knock-down (left). Counting of β-gal positive cells based on staining images (right). HCT p53+/- cells were transfected with Si-Lamin A (1.5 μg/ml, 48 hr). After transfection, cells were treated with Etoposide (10 μM) for different time periods (24 and 48 hr). Cells were then subjected to SA-β-gal staining. Boxes indicate magnified regions displayed in the right panel. *P<0.01. **<0.03. **(B)** Lamin A knock-down recovers DNA-damage induced growth retardation. Colony forming assay images show recovery of Etoposide induced growth retardation by Lamin A knock-down in p53 wild-type HCT116 cells. HCT p53-/- cells were transfected with Si-Lamin A (1.5 μg/ml, 48 hr) following Etoposide treatment (10 μM) at different days (1-5 Days). After treatment, cells were subjected to colony forming assay. **(C)** Knock-down of Lamin A does not affect recovery of Nutlin-3 induced growth retardation in p53 null cells. p53 null HCT116 cells (HCT p53-/-) did not show recovery of Nutlin-3 induced growth retardation by Si-Lamin A. HCT p53-/- cells were transfected with Si-Lamin A (1.5 μg/ml, 48 hr) following Nutlin-3 (1 μM) treatment for different days (1-5 Days). After treatment, cells were subjected to colony forming assay. **(D)** Lamin A overexpression is sufficient to induce p16 expression. Western blot images show that Lamin A overexpression could elevate p16 expression in p53 negative cells, similar to p53 transfection effect. HCT p53-/- cells were transfected with GFP-Lamin A and p53 (1.5 μg/ml, 48 hr). After transfection, cell extracts were analyzed by western blot using specific antibodies. **(E)** Lamin A overexpression increases p16 expression. IF images show that exogenous Lamin A increases p16 expression. HCT p53-/- cells were transfected with GFP-Lamin A and p53 (1.5 μg/ml, 48 hr). After transfection, cells were IF staining with p16 (Red), p53 (Green)/GFP, and counter staining with DAPI (Blue). **(F)** Lamin A overexpression decreases H3K9me3 expression. IF images show that exogenous Lamin A decreases H3K9me3 expression. HCT p53-/- cells were transfected with GFP-Lamin A and p53 (1.5 μg/ml, 48 hr). After transfection, cells were IF stained with H3K9me3 (Red), p53 (Green)/GFP, and counter stained with DAPI (Blue). **(G)** Cells with H3K9me3 positive (left) and intensity (right) were counted in (F). *P<0.01. **(H)** Lamin A overexpression is sufficient to induce cellular senescence. As much as p53 transfection, Lamin A transfection showed β-gal stained cells in p53 negative cells (above). Cells with β-gal positive were counted (below). HCT p53-/- cells were transfected with GFP-Lamin A and p53 (1.5 μg/ml, 48 hr). After transfection, cells were subjected to SA-β-gal staining. *P<0.01.

**Supplementary Figure 13.** Direct interaction of Lamin A/progerin with BMI-1/MEL-18. **(A)** MEL-18/BMI-1 does not bound to C terminal of Lamin A or progerin. GST pull-down assays show binding of FLAG tagged MEL-18/BMI-1 with Lamin A/progerin recombinant protein (LMN; Lamin A, PRG; Progerin). PPT: Pellet, Sup: Supernatant. LE and SE indicated long-exposure and short exposure, respectively. **(B)** MEL-18 bound to middle region of Lamin A. FLAG-tagged MEL-18 lysates were mixed with His-tagged Lamin fragment recombinant proteins (His-Lamin A N, His-Lamin A M) and then subjected to immunoprecipitation assay. MEL-18 especially binds to Lamin A N terminal region. PPT: Pellet, Sup: Supernatant. **(C, D)** Binding of MEL-18/BMI-1 with Lamin A/progerin. Immunoprecipitation assays show that both FLAG-tagged BIM1/MEL-18 (FLAG-MEL-18/BMI-1) bind to GFP-tagged LaminA/progerin (GFP-Lamin A/Progerin). PPT: Pellet, Sup: Supernatant. **(E)** MEL-18/BMI-1 expression is decreased by Lamin A/progrin. Exogenous FLAG tagged MEL-18/BMI-1 (FLAG-MEL-18/BMI-1) are downregulated by co-transfected GFP-tagged Lamin A/progerin (GFP-Lamin A/Progerin). HEK293 cells were co-transfected with vectors (1.5 μg/ml, 48 hr each). After transfection, cell extracts were analyzed by western blot using specific antibodies. Actin is used as the loading control.

**Supplementary Figure 14.** p53 suppresses expression of MEL-18 and BMI-1. **(A)** Nutlin-3 dependent p53 stabilization increases expression of Lamin A/C and p16 but decreases expression of BMI-1 irrespective of p53 transcriptional inhibition. Only NOXA expression is decreased in the presence of PTF-α. HCT p53+/- cells were treated with Nutlin-3 (1 μM) for different time periods (24 and 48 hr) with or without PFT-α (20 μM). After treatment, cell extracts were subjected to western blot using specific antibodies. **(B)** p53 decreases MEL-18/BMI-1 expression. Immunofluorescence images show decreased expression of MEL-18/BMI-1 by co-transfection with p53 in p53 negative PC3 cells. PC3 cells were transfected with FLAG-tagged MEL-18, BMI-1 (FLAG-MEL-18/FLAG-BMI-1) and co-transfected with p53 (1.5 μg/ml, 48 hr). After transfection, cells were IF stained with FLAG (Red), p53 (Green), and counter stained with DAPI (Blue). **(C-E)** BMI-1 expression was decreased by Nutlin-3 treatment in HCT p21-/- (C), PUMA-/- (D), and Bax-/- cells (E). BMI-1 suppression by Nutlin-3 induced p16 increase. Cells were treated with Nutlin-3 (1 μM) for different time periods (24 and 48 hr) with or without PFT-α (20 μM). After treatment, cell extracts were subjected to western blot using specific antibodies. **(F-H)** p53 induction by UV reduces BMI-1 and increases p16/INK4A expression in HCT116 p21-/- (F), PUMA (G), and Bax-/- (H) isogenic cell lines. Each cell line was exposed to UV irradiation (70 J/m^2^) and subjected to western blot analysis.

**Supplementary Figure 15.** p53 suppresses MEL-18 and BMI-1 via Lamin A/C. **(A, B)** Western blot analyses show decreased expression of MEL-18/BMI-1 by co-transfected p53 in p53 negative HCT p53-/- (A), PC and H1299 cells (B). Actin is used as a loading control. Vectors (1.5 g/ml, 48 hr each) were transfected in p53 null cells and cell extracts were analyzed by western blot using specific antibodies. **(C)** Western blot images of Si-Lamin A in HCT p53-/- cell. Si-Lamin A (2.5 μg/ml) was transfected in HCT p53-/- cells and extracts were analyzed by western blot. **(D)** BMI-1 suppression by p53 is abrogated by Lamin A knock-down. p16 is increased by p53 through Lamin A/C induction. However, Si-Lamin A abrogates p16 increase. HCT p53-/- were transfected with Si-Lamin A (2.5 μg/ml, 48 hr) and co-transfected with p53 at different concentrations (1-3 μg/ml, 48 hr). Cell extracts were analyzed by western blot. **(E, F)** Treatment with Adriamycin (E) or Nutlin-3 (F) increases p16 expression at transcriptional level while Si-Lamin A abrogates these effects. HCT p53+/- cells were transfected with Si-Lamin A (2.5 μg/ml, 48 hr). After transfection, Adriamycin (2 μg/ml) or Nutlin-3 (1 μM) was used for treatment at different time periods (3, 6 and 12 hr). After transfection and chemical treatment, RT-PCR was performed. **(G, H)** Increase of p16 by p53 induction is not affected by PFT-α. Exogenous p53 transfection (G) in HCT p53-/- or endogenous p53 stabilization by Nutlin-3 (H) increased p16 expression, but Lamin A knock-down abrogated these effects independent of PFT-α. HCT p53-/- cells were co-transfected with p53 (1.5 μg/ml, 48 hr) and Si-Lamin A (2.5 μg/ml, 48hr) following chemical treatment with PFT-α (20 μM, 24 hr) (G). HCT p53+/- cells were transfected with Si-Lamin A (2.5 μg/ml, 48hr) following chemical treatment with Nutlin-3 (1 μM, 48 hr) and PFT-α (20 μM, 24 hr) (H). After treatment, cells were analyzed by RT-PCR.

**Supplementary Figure 16.** Progerin suppresses BMI-1 more obviously than Lamin A in a p53 dependent manner. **(A)** Proteasomal inhibition restores p53 induced BMI-1 suppression. p53 transfection suppresses BMI-1. However, BMI-1 expression is restored by MG132 and ALLN. Autophagy related chemicals (Bafilomycin A; BFA, 3-Methyladenine; 3-MA and Rapamycin) do not affect restoration of BMI-1 suppression by p53. HCT p53-/- cells were transfected with p53 (1 μg/ml, 48 hr). After transfection, cells were treated with chemicals MG132 (10 μM, 12 hr), ALLN (5 μg/ml, 12 hr), BFA (5 μg/ml, 24 hr), 3-MA (1 μM, 24 hr) and Rapamycin (50 μg/ml, 24 hr). LE and SE indicated long-exposure and short exposure, respectively. **(B)** Proteasomal inhibition restores Lamin A/progerin induced BMI-1 suppression. Exogenous Lamin A/progerin (GFP-Lamin A/Progerin) suppresses BMI-1 expression which is recovered by MG132 and ALLN proteasome inhibitor. Autophagy inhibitor BFA does not recover the suppression of BMI-1. HCT p53+/- cells were transfected with exogenous GFP tagged Lamin A and Progerin (1.5 μg/ml, 48 hr) following chemical treatment with MG132 (10 μM, 12 hr), ALLN (5 μg/ml, 12 hr) and BFA (5 μg/ml, 24 hr). After treatment, cell extracts were analyzed by western blot. LE and SE indicated long-exposure and short exposure, respectively. **(C)** Proteasomal inhibitor downregulates p53 induced p16/INK4A increase. PCR images show that proteasomal inhibitors suppress p53 induced p16/INK4A. HCT p53-/- cells were transfected with p53 (1 μg/ml, 48 hr) following chemical treatment with MG132 (10 μM, 12 hr), ALLN (5 μg/ml, 12 hr), BFA (5 μg/ml, 24 hr), 3-MA (1 μM, 24 hr), and Rapamycin (50 μg/ml, 24 hr). After chemical treatment, RT-PCR was performed. **(D)** Lamin A/Progerin induced p16 luciferase activity is abrogated by proteasome inhibition. Luciferase activity shows upregulation of p16 expression by Lamin A/progerin overexpression in HCT p53+- cells. p16 upregulation by progerin was more remarkable than Lamin A in HCT p53+/-. *P < 0.03, **P <0.001. **(E, F)** Immunofluorescence images showing MEL-18/BMI-1 suppression by Lamin A/progerin. p53 isogenic HCT cells were transfected with FLAG tagged BMI-1 with GFP-tagged Lamin or Progerin (1.5 μg/ml, 48 hr). After transfection, cells were immunofluorescence stained with FLAG (Red), counter stained with DAPI (Blue), and detected for GFP (Green).
